# Determining the impact of alternative splicing events on transcriptome dynamics

**DOI:** 10.1186/1756-0500-1-94

**Published:** 2008-10-24

**Authors:** Emmanuelle Wilhelm, François-Xavier Pellay, Arndt Benecke, Brendan Bell

**Affiliations:** 1 RNA Group. Département de microbiologie et d'infectiologie, Faculté de médecine et sciences de la santé, Université de Sherbrooke, 3001 12e ave Nord, Sherbrooke, Québec J1H 5N4, Canada; 2 Institut des Hautes Études Scientifiques & Institut de Recherche Interdisciplinaire – CNRS USR3078 – Université Lille1; 35 route de Chartres; 91440 Bures sur Yvette, France

## Abstract

**Background:**

The complete sequencing of the human genome and its subsequent analysis revealed a predominant role for alternative splicing in the generation of proteome diversity. Splice switching oligonucleotides (SSOs) are a powerful and specific tool to experimentally control alternative splicing of endogenous messenger RNAs in living cells. SSOs also have therapeutic potential to treat diseases that are caused by aberrant splicing. The assignment of biological roles to alternative splicing events of currently unknown function promises to provide a largely untapped source of potential new therapeutic targets. Here we have developed a protocol that combines high sensitivity microarrays with the transfection of SSOs to monitor global changes in gene expression downstream of alternate, endogenous splice events.

**Results:**

When applied to a well-characterized splicing event in the Bcl-x gene, the application of high sensitivity microarrays revealed a link between the induction of the Bcl-xS isoform and the repression of genes involved in protein synthesis.

**Conclusion:**

The strategy introduced herein provides a useful approach to define the biological impact of any given alternative splicing event on global gene expression patterns. Furthermore, our data provide the first link between Bcl-xS expression and the repression of ribosomal protein gene expression.

## Background

The completion of the human genome sequence [1,2] and subsequent analysis of the annotated genome [3] have revealed that the process of alternative splicing plays a key role in the generation of proteome diversity. Alternative splicing decisions control many biological processes ranging from sex determination in fruit flies to programmed cell death in human cells [4]. Recent estimates suggest that at least 74% of human genes undergo alternative splicing [5]. Moreover, a rapidly growing body of evidence shows that defects in alternative splicing of mRNA are an important cause of human disease [6-8]. Strikingly, although alternative splicing is pervasive within human genes, the functions of the vast majority of alternative splicing events remain unknown [3].

Technical advances in the application of antisense RNA oligonucleotides to manipulate alternative splicing either in cultured cells for functional genomic studies, or potentially in patients for therapeutic purposes, are opening unprecedented avenues to characterize the function of new alternative splicing events and eventually to exploit these functions clinically. These splice switching oligonucleotides (SSOs) can be used to induce loss of function or, when modified to bind cellular proteins, to induce gain of function by directing splicing regulatory factors to target a given splicing event [6,8,9].

The use of genome-wide microarrays in conjunction with SSO is an underutilized but potentially powerful method to determine the functions of alternative splicing events. Here we have recorded transcriptome dynamics during SSO-mediated induction of a well-characterized Bcl-xS splice variant using high-sensitivity microarrays. The results reveal a new link between Bcl-xS expression and the repression of ribosomal protein gene expression.

## Methods

Cell culture conditions, molecular biology techniques and an extensive description of SSO-coupled transcriptome analysis are provided [see Additional file 1].

## Results

**Figure 1:**
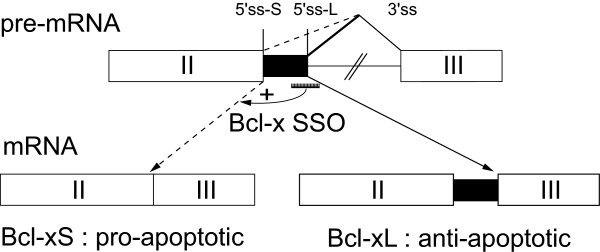
**Splice-switching oligonucleotides act on the endogenous Bcl-x gene to enforce splice site choice of the Bcl-xS**. The region of the Bcl-x pre-mRNA that includes two alternative 5' splice sites (SSs) that produce either the constitutive long (L) splice variant or the alternative short (S) splice variant is schematically depicted. Selection of an intron-proximal L 5' splice site (SS) results in production of the Bcl-xL isoform (at right) whereas the selection of the proximal S 5' SS results in the production of the Bcl-xS isoform (at left). The SSO oligonucleotide that base pair with the major L splice site forces splicing from the distal 5' SS and induces expression of the endogenous Bcl-xS isoform (at left). The Bcl-xL gene product has anti-apoptotic activity while the Bcl-xS gene product possesses pro-apoptotic activity.

The Bcl-x gene is alternatively spliced to produce the major Bcl-xL (long) isoform with anti-apoptotic activity and a minor Bcl-xS (short) isoform that can promote apoptosis [10]. A distal alternative 5' splice site (SS) is used to produce a short Bcl-xS isoform that possesses pro-apoptotic activity. We chose as a model system the Bcl-x gene to study downstream effects of inducing the endogenous Bcl-xS splice variant because: 1) splice-switching oligonucleotides were first developed and tested to enforce splice site selection of the endogenous Bcl-x gene [11,12], 2) the biological consequences of inducing the pro-apoptotic Bcl-xS isoform are reasonably well-characterized, and 3) the Bcl-x gene is a potential target for SSO-based anti-cancer therapies. We used synthetic 2'-*O*-methyl-modified oligoribonucleoside phosphorothioate SSO as previously described by Kole and colleagues [12,13] to enforce splicing of Bcl-xS as schematised in Figure 1.

**Figure 2:**
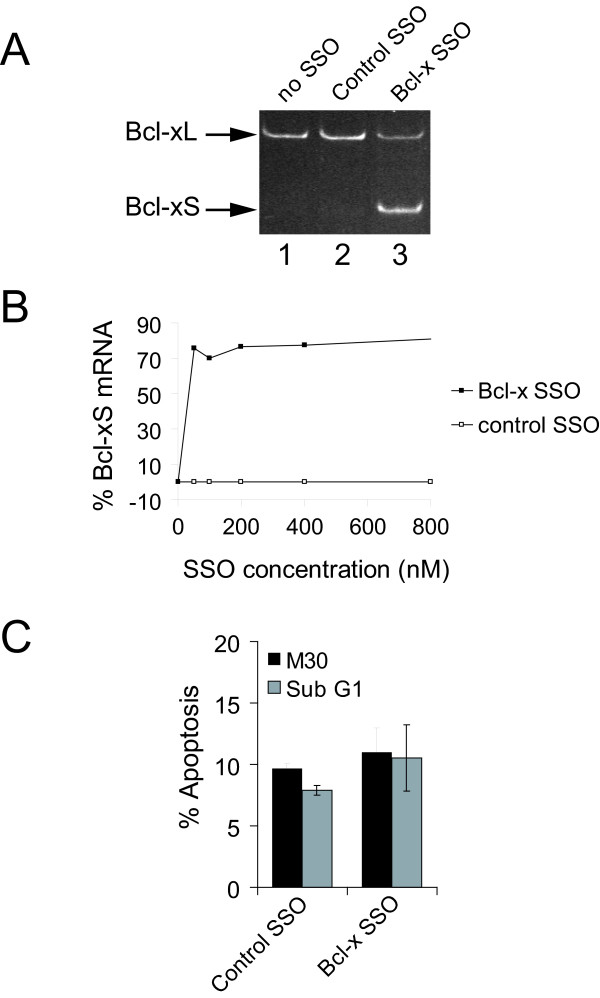
**Specific control of endogenous Bcl-xS splicing by modified antisense RNA oligonucleotides in living cells**. (A) Antisense RNA oligonucleotide induces endogenous Bcl-xS mRNA expression. HeLa cells were transfected with 200 nM oligonucleotide directed against the major Bcl-xL splice site (Bcl-x SSO) or a scrambled control oligonucleotide (Control SSO). 24 hours post-transfection total RNA was isolated and subjected to RT-PCR with primers that amplify both the Bcl-xL and the alternative Bcl-xS mRNAs. (B) Concentration-dependent antisense mediated induction of Bcl-xS mRNA expression. HeLa cells were transfected with antisense RNA oligonucleotides as in A. RT-PCR was performed as in A. PCR products were separated by microfluidity and quantified using a 2100 Agilent bioanalyzer. The ratio of Bcl-xS mRNA over total Bcl-x mRNA is expressed on the y-axis. The values from cells treated with scrambled control SSO (open squares), or Bcl-X SSO (black squares) are shown. (C) The effect of endogenous Bcl-xS on apoptosis in HeLa cells. HeLa cells were transfected as in A. Cells were harvested 24 hours post-transfection and percentage of apoptotic cells was analyzed by flow cytometry using monoclonal antibodies that detect caspase cleaved cytokeratin-18 (M30; black bars), or by measuring Sub G1 DNA content (grey bars). Error bars represent the standard deviation of three independent transfections.

HeLa cervical carcinoma cells were transfected with an SSO that anneals to the major 5' SS of exon II of the Bcl-x gene to switch splicing to the minor 5' SS (Bcl-x SSO, Figure 1). Lipofectamine was selected as the transfection reagent as it has previously been demonstrated to provide efficient delivery of 2'-O-methyl (2'-O-Me) phosphorothioate oligonucleotides into the nucleus of HeLa cells [14]. The impact of transfection of Bcl-x SSO on the expression of the alternative Bcl-xS splice variant was analyzed by RT-PCR with primers flanking the alternative splicing event. In untreated HeLa cells the levels of Bcl-xS mRNA were very low (Figure 2A, lane 1). Treatment of HeLa cells with a control SSO of scrambled sequence did not alter the levels of Bcl-xS mRNA (Figure 2A, lane 2), indicating the absence of non-specific effects of transfection of SSOs on Bcl-xS, consistent with previous reports employing an independent scrambled SSO [12] or 5 nucleotide mismatched SSO [11]. The transfection of the Bcl-x SSO caused, as previously reported, a strong shift from the predominant expression of Bcl-xL towards the expression of Bcl-xS (Figure 2A, lane 3). The levels of induction of endogenous Bcl-xS by SSO were analyzed quantitatively by transfection of increasing concentrations of the Bcl-x SSO into HeLa cells followed by analysis of the ratio of endogenous Bcl-xS/Bcl-xL+xS mRNA using Agilent LabChips. The percentage of Bcl-xS/Bcl-xL+xS in the presence of Bcl-x SSO reached 77% with treatment of cells with 200 nM SSO and higher concentrations produced very little further increase in the Bcl-xS/Bcl-xL+xS ratio (Figure 2B). In contrast, no change in the Bcl-xS/Bcl-xL+xS ratio was seen with the negative control SSO of random sequence at any concentration used (Figure 2B). Based on the above results we selected treatment with 200 nM Bcl-x SSO as a robust method to enforce splice site selection of endogenous Bcl-xS for microarray studies.

Treatment of cells with Bcl-xS-inducing SSOs can either sensitize cells to apoptotic stimuli, or induce apoptosis outright depending on the cell type and the levels of expression of Bcl-x [11-13]. In order to correlate changes in gene expression induced by Bcl-xS expression with the phenotypic outcome it was important to measure the rates of apoptosis in our experimental system. To analyze the impact of shifting the expression of endogenous Bcl-x from the long to the short isoform on apoptosis, the percentage of apoptotic cells was analyzed by flow cytometry using monoclonal antibodies that detect caspase cleaved cytokeratin-18, or by measuring Sub G1 DNA content by staining with propidium iodide. HeLa cells treated with Bcl-x SSO did not show a statistically significant increase in levels of apoptosis, in line with the previous results of Mercatante et al. in HeLa cells [13]. Therefore although Bcl-xS mRNA expression is strongly induced in HeLa cells, this change is not accompanied by cell death through apoptosis.

**Figure 3:**
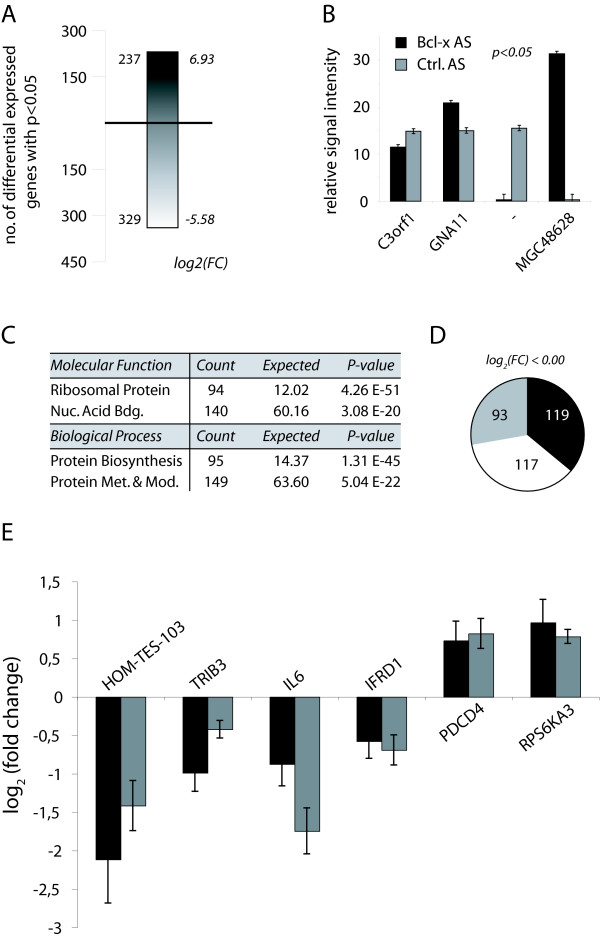
**Endogenous Bcl-xS expression orchestrates a cellular gene expression program linked to the protein synthesis machinery**. (A) Transcriptome analysis following Bcl-x SSO in HeLa cells. Expression levels of mRNAs from Bcl-x AS-treated cells were compared to control oligonucleotide-treated HeLa samples 18 hours post SSO transfection by genome-wide microarray analysis using three independent biologic replicates each. The absolute number of probes detecting statistically significant (p < 0.05) up- or down-regulation following splice-site selection is shown to the left of the bar; the maximum positive or negative logarithmic (base two) fold-change is shown to the right. The black gradient indicates positive, and the white gradient negative fold changes in expression. (B) The relative, normalized, averaged chemiluminescence signal intensities for the least repressed (C3orf1), the least induced (GNA11), the most repressed ("-"; no annotation information available), and the most induced (MGC48628) gene when comparing the Bcl-x AS oligonucleotide (black bars) to the control AS oligonucleotide (grey bars) at a statistical significance threshold of p < 0.05 as well as the standard deviations over those signals are compared. (C) Specific biologic processes and molecular functions are statistically significantly (p < 0.01) overrepresented in the Bcl-xS-transcriptome. "Count" indicates the number of probes within the Bcl-x dataset that can be attributed to the corresponding ontology term, "Expected" is the number of probes one would expect to find for a given ontology term in a random dataset of identical size. (D) Pie-chart analysis of genes down-regulated by endogenous Bcl-xS SSO illustrating the number of genes associated with the ontology term "molecular function: Ribosomal Protein" (grey), and "molecular function: Nucleic Acid Binding" (black) as a fraction of all genes down-regulated by endogenous Bcl-xS SSO. (E) Verification of gene expression changes by quantitative real-time RT-PCR. HeLa cells were transfected with antisense oligonucleotides that induce endogenous Bcl-xS expression. 18 hours post-transfection total RNA was isolated from cells and expression level changes were analyzed by quantitative real-time PCR (grey bars) and compared with changes in expression from microarray measurements (black bars) as logarithmic base-two fold changes. Error bars indicate standard deviation of three independent transfections. The Pearson correlation coefficient calculated for the correlation between the microarray data and the qPCR is 0.872.

**Figure 4:**
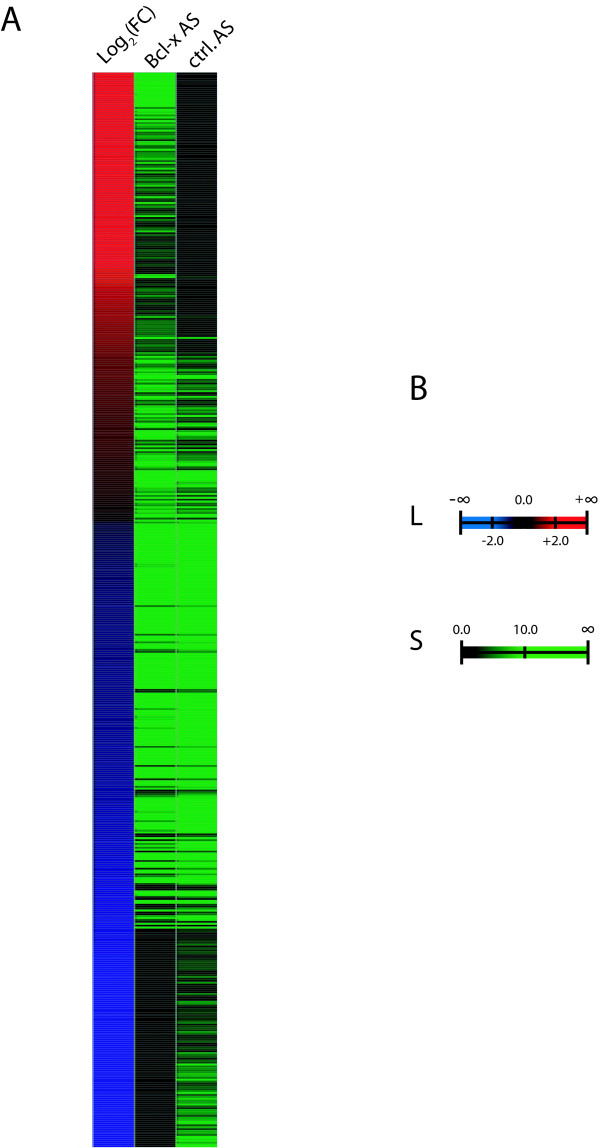
**Transcriptome analysis**. (A) A Heat-map for the Bcl-x AS versus control AS subtraction profile is shown. Note that the non-normalized signal intensities for the probes revealing statistically significant changes in gene expression (p < 0.05) are represented by a black (low signal) to green (high signal) gradient. The probes are ordered in a logarithmic (base-two) fold-change descending order ("log_2_(FC)") which is displayed as a red (highly up-regulated) to black to blue (highly down-regulated) gradient. (B) A color code legend for the data displayed in (A) is given.

To study the impact of Bcl-xS induction on gene expression, we decided to record the downstream transcriptome dynamics using pan-genomic 60mer oligonucleotide microarrays and chemiluminescence detection chemistry, a combination that has previously been shown to provide increased signal dynamic range and higher sensitivity when compared to traditional microarray technologies [15,16]. The microarrays used represent 27,868 fully annotated and verified human genes. Biologic triplicate recordings for both control and Bcl-xS SSO transfected HeLa cells were acquired following total RNA purification and reverse transcriptase labeling as detailed in the methods section [see Additional file 1]. After image processing, and primary data analysis, we calculated the logarithmic (base 2) fold-change for all represented genes and determined their level of statistical significance according to standard methods. We found a total of 556 genes [see additional file 2] to be differentially expressed with a p-value inferior to 0.05 when comparing the transcriptome from control and Bcl-xS SSO transfected HeLa cells (Figure 3A &4). Slightly more genes are repressed by Bcl-x SSO than are induced as 329 or 59% of the genes show a decrease in expression following splice-site shifting in Bcl-x. The quality of the transcriptome recordings and their analysis can be appreciated from comparing the signal intensities of the least and most induced or repressed genes (Figure 3B). In the case of the chromosome 3 open-reading frame 1 as little as a 1.2 fold-change is readily detected. We then analyzed the differentially expressed gene list for emergent properties using a meta-analysis of the available annotation information for statistically significant (p < 0.01) over-represented molecular function and biologic process ontologies. Indeed, for each category, two ontologies are highly significantly enriched (Figure 3C). Note that the molecular function and biologic process ontologies are not mutually exclusive, quite to the contrary, the signals in both categories are generated by essentially the same subset of genes [see Additional files 3, 4, 5 and 6], and point towards a highly relevant enrichment of the protein synthesis apparatus in the Bcl-xS regulated gene set. Further analysis revealed that the expression levels of the majority of genes belonging to the molecular functions singled out through the meta-analysis are reduced following a shift in the cellular equilibrium towards Bcl-xS (Figure 3D). For example, of 94 genes significantly regulated with the molecular function ontology being ribosomal protein genes, 93 are repressed (Figure 3C &3D). Finally, for a selected number of Bcl-xS targets we performed independent biological triplicate quantitative RT-PCR analysis to demonstrate reproducibility of our transcriptome measurements with an independent technology (Figure 3E). As expected, the qPCR results confirm the microarray data for all tested genes (Figure 3E). The Pearson correlation coefficient for the correlation between the microarray data and the qPCR is 0.872, providing a statistical measure that reinforces the validity of the microarray data. We therefore have demonstrated the feasibility of using high sensitivity transcriptome analysis as a tool for defining the outcome of alternative splicing events on global gene expression patterns.

## Conclusion

Here we have combined splice-switching oligonucleotides (SSOs) with high-sensitivity genome-wide microarrays to reveal the impact of shifting the expression of the endogenous Bcl-xL splice variant towards the Bcl-xS splice variant. To our knowledge, only a single study attempted to address the impact of SSO on gene expression patterns using microarrays [17]. In that study, no statistically significant impact of Bcl-xS SSO on specific cellular functions or pathways was uncovered. Here we demonstrate a statistically significant impact of Bcl-xS SSOs on the repression of ribosomal protein-encoding genes and genes that code for nucleic acid binding proteins. Several factors could contribute to the different outcome of our study when compared to the earlier study [17]. For example, different cell lines can give different transcriptome footprints, and we have employed HeLa whereas Mercatante et al. employed MCF7 and PC3 cells. Moreover the time of SSO treatment in our study was 18 hours versus 36 hours in that of Mercatante et al., a difference that could influence the number of primary versus secondary effects of the SSO. Here we have combined for the first time SSO with second-generation microarrays that possess substantially increased sensitivity and genome coverage and thus provide improved detection of statistically significant changes in specific gene ontologies. The capacity to detect specific cellular pathways that are impacted upon SSO represents an advance that can provide new biological insights into the function of alternative splicing events. Our study has focused on the transcriptome signature induced by Bcl-x SSO in the HeLa cell model system, however the methodology we describe should also be applicable to any alternative splicing event in any cell line that responds efficiently to SSO transfection. Genome-wide technologies have revealed the pervasive role of alternative splicing in proteome diversity, yet the functions of the vast majority of splice variants remain unknown [3]. The merging of SSO and second-generation microarray technology that we present here provides a powerful and broadly applicable methodology to decipher the biological functions that are controlled by alternative splicing events.

The transcriptome footprint caused by the induction of endogenous Bcl-xS results in a dramatic suppression of genes involved in protein synthesis, particularly ribosomal protein encoding genes (Figure 3C). Since we do not observe a strong apoptotic response in cells treated with Bcl-x SSO (Figure 2C), we conclude that the measured decreases in the expression of genes related to protein synthesis cannot alone induce apoptosis. Bcl-xS has been characterized as a pro-apoptotic factor that is localized largely to the mitochondria [18]. An impact of Bcl-xS-inducing SSO on the suppression of genes that participate in protein synthesis was therefore an unanticipated outcome. Boone-Unge and colleagues [19] have recently reported a biological link between the inhibition of protein translation and Bcl-xS using a completely unrelated approach. Their data showed that the translational inhibitor emitine causes a significant increase in the cellular levels of Bcl-xS mRNA. It is therefore conceivable that a feedback loop between Bcl-xS expression and translational inhibition exists. Further work will be required to confirm the hypothesis of such a feedback mechanism.

In summary, we report here a protocol that enables the measurement of the impact of endogenous alternative splicing events on transcriptome dynamics. When applied to the induction of Bcl-xS mRNA by splice-switching oligonucleotides we demonstrate a statistically significant suppression of ribosomal protein encoding genes. The approach applied here should accelerate efforts to define the biological roles of the vast numbers of uncharacterized alternative splice variants within the human genome. Finally, the approach we describe can also be applied to the analysis of the mechanism of action of clinically useful SSOs.

## Competing interests

The authors declare that they have no competing interests.

## Authors' contributions

EW performed the experiments except the microarray experiments. FXP analyzed microarray data. AB designed and performed the microarray experiments and their statistical analysis. BB designed the experiments and performed data analysis. EW, AB and BB wrote the paper. All authors read and approved the final manuscript.

## Supplementary Material

Additional file 1 Detailed Methods. A detailed description of the methods used including cell culture conditions, molecular biology techniques and an extensive description of SSO-coupled transcriptome analysis. Click here for file

Additional file 2 An ascii tab-delimited file is provided containing the listing of the probe IDs (PROBE), the log2 fold-changes (LOG_Q), the log2 fold change estimated variance (VAR), the P-value (P), as well as several gene identifiers, for the 565 selected probes from this analysis. Click here for file

Additional file 3 An ascii tab-delimited file is provided containing the listing of the probe IDs (PROBE), the log2 fold-changes (LOG_Q), the log2 fold change estimated variance (VAR), the P-value (P), as well as several gene identifiers, for the subset of selected probes mapping to the Nucleic Acid Binding ontology term used for the meta-analysis. Click here for file

Additional file 4 An ascii tab-delimited file is provided containing the listing of the probe IDs (PROBE), the log2 fold-changes (LOG_Q), the log2 fold change estimated variance (VAR), the P-value (P), as well as several gene identifiers, for the subset of selected probes mapping to the Protein Biosynthesis ontology term used for the meta-analysis. Click here for file

Additional file 5 An ascii tab-delimited file is provided containing the listing of the probe IDs (PROBE), the log2 fold-changes (LOG_Q), the log2 fold change estimated variance (VAR), the P-value (P), as well as several gene identifiers, for the subset of selected probes mapping to the Protein Metabolism and Modification ontology term used for the meta-analysis. Click here for file

Additional file 6 An ascii tab-delimited file is provided containing the listing of the probe IDs (PROBE), the log2 fold-changes (LOG_Q), the log2 fold change estimated variance (VAR), the P-value (P), as well as several gene identifiers, for the subset of selected probes mapping to the Ribosomal Protein ontology term used for the meta-analysis. Click here for file
