# Supplementary material for: Determining the impact of alternative splicing events on transcriptome dynamics
Source: BMC Res Notes. 2008 Oct 24;1:94. doi: 10.1186/1756-0500-1-94 (PMC2584107; doi:10.1186/1756-0500-1-94)
Supplement: Additional file 1 — Detailed Methods. A detailed description of the methods used including cell culture conditions, molecular biology techniques and an extensive description of SSO-coupled transcriptome analysis. [file 1756-0500-1-94-S1.doc]

**Cell culture**

HeLa cells were grown in DMEM containing 2.5% CS and 2.5% FCS.

**Transfections**

Oligonucleotides were transfected with lipofectamine 2000 (Invitrogen) as a delivery agent (1.6 µl/ml) according to the manufacturer’s recommendations. 2’-O-methyl-oligoribonucleoside phosphorothioate antisense 20-mers were from Sigma-Proligo. “Control SSO” 5’-AUGGCCUCGACGUGCGCGCU-3’ is a scrambled oligo used as a negative control. “Bcl-x SSO” 5’-ACCCAGCCGCCGUUCUCC-3’ targets the 5’-splice site of Bcl-xL [1]. All transfections were performed in OptiMEM medium (Invitrogen).

**RT-PCR**

Total RNA was isolated from cells using Trizol (Invitrogen) according to the manufacturer’s recommendations. 1µg of total RNA was reverse transcribed using AMV-RT (Roche). 1/10 of the total cDNA was used per PCR reaction : 95°C, 3 min; 25 cycles of 94°C for 1 min, 58°C for 45 sec, 68°C for 50 sec; final extension at 68°C for 5 min with the following oligonucleotide pairs. For Bcl-x; forward 5’-TCATTTCCGACTGAAGAGTGA-3’ and reverse 5’-ATGGCAGCAGTAAAGCAAGCG-3’

**Apoptosis assays**

Detection of caspase cleaved cytokeratin-18 by flow cytometry was performed using Cytodeath reagent (Roche) according to the manufacturer’s recommendations. Flow cytometric analysis of sub-G1 DNA content was performed as described [2].

**Microarray Analysis of Gene Expression**

Transcriptome Acquisition.

Total RNA was extracted 18 hours post SSO transfection with RNeasy kit (Qiagen) and analyzed using ABI Human Whole Genome Survey Arrays v1.0 arrays (Prod. No.: 4359030), containing 31,700 60-mer oligonucleotide probes representing a set of 27,868 individual annotated human genes. Chemiluminescence detection technology is used to detect as little as a femtomole of expressed mRNA. One single round of linear amplification was performed from total RNA according to the Applied Biosystems RT-IVT (Applied Biosystems, ProdNo: 4339628) protocol using 2µg of total RNA. cDNA synthesis, *in vitro* transcription and labeling, fragmentation, hybridization, staining, and scanning were performed as directed by the supplier (Applied Biosystems, ProdNo: 4346875).

Transcriptome Data Analysis.

Applied Biosystems Expression Array System Software v1.1.1. (ProdNo: 4364137) has been used to acquire the chemiluminescence and fluorescence images and primary data analysis. We renormalized the resulting data according to the logarithmic signal median once more after having removed probes for which the Applied Biosystems Software has set flags equal to or greater than 2^12^, indicating compromised measurements (as recommended by Applied Biosystems). Log_2_ subtractions were determined using averages over the weighted individual signal values. The weights are anti-proportional to the corresponding coefficient of variation. For these inter-assay comparisons the NeONORM method was used for normalization using sensitivity parameter *k*=0.20 [3]. P-values were determined using a mixed model ANOVA controlling false discovery rate at 5%. The two models (intensity-specific min/max mean error variance and actual error variance) are determined for every experiment based on the mixture distribution analysis for Applied Biosystems AB1700 data [4]. Multiple probes for a single gene, cross-reactivity of a single probe to several genes, as well as the resolution of probe-ID annotations were done according to the standards defined previously [5]. Probes with p-values of less than 0.05 were compiled [see additional file 2] and available online at http://www.iri.cnrs.fr/seg/WilhelmEtAl2008BMCrn.zip. Gene Ontology (GO) and KEGG annotations were analyzed using the Panther Protein Classification System (http://www.pantherdb.org) to identify functional annotations that were significantly (p<0.01) enriched in the differentially expressed gene set when compared to the whole set of genes present on the ABI microarray. P-values are determined using a binominal distribution and a null hypothesis of a random set of genes with identical size. A Bonferroni correction for multiple testing was applied. The probes from the above selection of statistically significantly regulated genes mapping to any one of the analyzed ontologies are also provided [see Additional files 3, 4, 5 and 6], equally available from http://www.iri.cnrs.fr/seg/WilhelmEtAl2008BMCrn.zip.

The microarray data described here were deposited in the Gene Expression Omnibus database (http://www.ncbi.nlm.nih.gov/geo/) under accession number: [GSE9851](http://www.ncbi.nlm.nih.gov/projects/geo/query/acc.cgi?acc=GSE9851).

**Real time PCR**

Total RNA was extracted with RNeasy kit (Qiagen) and reverse transcribed as for RT-PCR (see above). Real-time PCR was performed on 10 ng of cDNA with 1.25 µl of 20x TaqMan® probes and 12.5 µl 2x TaqMan® Universal Master Mix (ABI) in a final 25µl reaction. Real-time PCR relative quantification assay was running for 2 min at 50 °C, 10 min at 95°C, followed by 40 cycles of 15 sec at 95 °C and 1 min at 60°C on an ABI 7500 system. Relative quantity of target genes was calculated using the comparative C_T_ (∆∆C_T_) method using β2microglobulin as internal control.

**References (Methods)**

1. Mercatante DR, Bortner CD, Cidlowski JA, Kole R: **Modification of alternative splicing of Bcl-x pre-mRNA in prostate and breast cancer cells. analysis of apoptosis and cell death**. *J Biol Chem* 2001, **276**(19):16411-16417.

2. Bell B, Scheer E, Tora L: **Identification of hTAF(II)80 delta links apoptotic signaling pathways to transcription factor TFIID function**. *Mol Cell* 2001, **8**(3):591-600.

3. Noth S, Brysbaert G, Benecke A: **Normalization using weighted negative second order exponential error functions (NeONORM) provides robustness against asymmetries in comparative transcriptome profiles and avoids false calls**. *Genomics Proteomics Bioinformatics* 2006, **4**(2):90-109.

4. Noth S, Brysbaert G, Pellay FX, Benecke A: **High-sensitivity transcriptome data structure and implications for analysis and biologic interpretation**. *Genomics Proteomics Bioinformatics* 2006, **4**(4):212-229.

5. Noth S, Benecke A: **Avoiding inconsistencies over time and tracking difficulties in Applied Biosystems AB1700/Panther probe-to-gene annotations**. *BMC Bioinformatics* 2005, **6**:307.
